# Short-Term Nitrogen Dioxide Exposure and Emergency Hospital Admissions for Asthma in Children: A Case-Crossover Analysis in England

**DOI:** 10.2147/JAA.S448600

**Published:** 2024-04-09

**Authors:** Weiyi Wang, John Gulliver, Sean Beevers, Anna Freni Sterrantino, Bethan Davies, Richard W Atkinson, Daniela Fecht

**Affiliations:** 1 UK Small Area Health Statistics Unit, MRC Centre for Environment and Health, Department of Epidemiology and Biostatistics, School of Public Health, Imperial College London, London, UK; 2 National Institute for Health and Care Research Health Protection Research Unit in Chemical and Radiation Threats and Hazards, School of Public Health, Imperial College London, London, UK; 3 Population Health Research Institute, St George’s, University of London, London, UK; 4 MRC Centre for Environment and Health, Environmental Research Group, School of Public Health, Imperial College London, London, UK; 5 National Institute for Health and Care Research Health Protection Research Unit in Environmental Exposures and Health, School of Public Health, Imperial College London, London, UK; 6 The Alan Turing Institute, London, UK

**Keywords:** asthma, nitrogen dioxide, children, hospital admissions, case-crossover

## Abstract

**Background:**

There is an increasing body of evidence associating short-term ambient nitrogen dioxide (NO_2_) exposure with asthma-related hospital admissions in children. However, most studies have relied on temporally resolved exposure information, potentially ignoring the spatial variability of NO_2_. We aimed to investigate how daily NO_2_ estimates from a highly resolved spatio-temporal model are associated with the risk of emergency hospital admission for asthma in children in England.

**Methods:**

We conducted a time-stratified case-crossover study including 111,766 emergency hospital admissions for asthma in children (aged 0–14 years) between 1st January 2011 and 31st December 2015 in England. Daily NO_2_ levels were predicted at the patients’ place of residence using spatio-temporal models by combining land use data and chemical transport model estimates. Conditional logistic regression models were used to obtain the odds ratios (OR) and confidence intervals (CI) after adjusting for temperature, relative humidity, bank holidays, and influenza rates. The effect modifications by age, sex, season, area-level income deprivation, and region were explored in stratified analyses.

**Results:**

For each 10 µg/m³ increase in NO_2_ exposure, we observed an 8% increase in asthma-related emergency admissions using a five-day moving NO_2_ average (mean lag 0–4) (OR 1.08, 95% CI 1.06–1.10). In the stratified analysis, we found larger effect sizes for male (OR 1.10, 95% CI 1.07–1.12) and during the cold season (OR 1.10, 95% CI 1.08–1.12). The effect estimates varied slightly by age group, area-level income deprivation, and region.

**Significance:**

Short-term exposure to NO_2_ was significantly associated with an increased risk of asthma emergency admissions among children in England. Future guidance and policies need to consider reflecting certain proven modifications, such as using season-specific countermeasures for air pollution control, to protect the at-risk population.

## Introduction

Epidemiological studies have provided a strong body of evidence for an association of short-term exposure to traffic-related air pollution with asthma exacerbations and hospitalisations in children.^1–5^ Children are particularly affected by the adverse health effects of air pollution because their lungs are still developing,^6^ they have a higher ventilation rate,^7^ and they are at a higher risk of viral respiratory infections.^8^

Most studies on the short-term health effects of air pollution rely on measurements obtained from routine monitoring sites assigned to a large number of study participants.^9^,^10^ This fails to account for spatial contrasts in air pollution exposure – varying from day to day – between individuals living in the same geographic region, which might lead to non-differential exposure misclassification and, consequently, attenuation of effect estimates towards the null. Advances in spatio-temporal modelling mean that studies can now predict daily exposure at a high spatial resolution, such as the place of residence.^11–14^

Nitrogen dioxide (NO_2_) is one of the main constituents of a group of reactive gases known as nitrogen oxides. The primary source of nitrogen oxides in England is road transport through the combustion of vehicle fuels. Therefore, the spatial distribution of NO_2_ tends to highest levels of concentrations in the vicinity of roads, especially in urban environments. Using an advanced spatio-temporal exposure model which we developed previously to predict daily NO_2_ concentrations for each residential postcode in Great Britain,^15^ we aimed to examine the association between short-term NO_2_ exposure and the risk of emergency hospital admissions for asthma in children across England. We used a case-crossover design and explored potential effect modifications by age, sex, season, area-level income deprivation, and region.

## Materials and Methods

We carried out a case-crossover analysis to compare residential NO_2_ exposure on the day of asthma-related emergency hospital admission with exposure on days without an event, and adjusted for time-variant confounders.

### Study Population and Outcome

We included all children aged 0–14 years who were admitted to a National Health Service (NHS) hospital in England between 1st January 2011 and 31st December 2015 with a primary diagnosis of asthma (International Statistical Classification of Disease 10th revision (ICD–10: J45, J46)). This age range was chosen based on established age brackets, which is consistent with previous studies on childhood asthma and air pollution.^16^,^17^ Daily emergency (non-elective) admissions were obtained from the Hospital Episode Statistics (HES) held by the UK Small Area Health Statistics Unit (SAHSU) at Imperial College London, provided by NHS England. Each admission record included the admission date, age, sex, and residential postcode of address at the time of admission. Each postcode represents the geographic centroid of approximately fifteen households.

The study was covered by national research ethics approval from the London–South East Research Ethics Committee (Reference 22/LO/0256). Data access to confidential patient information without consent was covered by the Health Research Authority - Confidentiality Advisory Group under Regulation 5 of the Health Service (Control of Patient Information) Regulations 202 (‘section 251 support’) (Reference 20/CAG/0008), which supports the application of confidential patient information in circumstances where it is not possible to use anonymised information and obtaining consent is not practical.

### Air Pollution Data

We modelled daily NO_2_ concentrations on a 25-metre grid using an advanced, validated spatio-temporal air pollution model that is described in detail elsewhere.^15^ Briefly, the model combines air pollution measurements from fixed sites from the UK Automatic Urban and Rural Monitoring Network (AURN, https://uk-air.defra.gov.uk/networks/network-info?view=aurn) and spatially highly resolved local-scale predictors derived using a Geographic Information System (including traffic load, length of major roads, and building volume around major roads) and temporal predictor variables (including daily estimates from a chemical transport model and daily meteorological characteristics) in a generalised additive mixed model framework. In the cross-validation against NO_2_ measurements from the monitoring sites, the model performed well and explained 63% of the variability in the daily averaged measured concentrations. Using the established model, we extracted the daily NO_2_ concentration for each patient’s post-code centroid.

### Confounders

By design, time-invariant confounders such as age, sex, and socioeconomic status are controlled for in the case-crossover study design as each case acts as its own control. We controlled for time-variant confounders, including meteorological data, public holidays, and influenza rates in accordance with previous studies.^18–20^ We computed the daily mean temperature and relative humidity for each patient postcode using ERA5-Land reanalysed hourly climate models from the European Centre for Medium-Range Weather Forecasts (https://www.ecmwf.int/en/era5-land) with a spatial resolution of 0.1° × 0.1°. To account for variations in air pollution levels associated with travel behaviours, we included bank holidays in England as a confounder. We obtained weekly influenza consultation rates from the Office for National Statistics (https://www.ons.gov.uk), reported as weekly influenza-like illnesses per 100,000 population by the Royal College of General Practitioners of England.

### Statistical Analysis

We used a time-stratified case-crossover study design.^21^ For each individual case, a “case day”, the day on which the outcome of interest occurs (ie date of admission), was matched to a set of “control days”, days on which the outcome of interest did not occur. Thus, each case was compared to itself, thereby controlling for time-invariant individual factors. We defined control days as those on the same day of the week and month (before and/or after the case day) to control for any potential time trends,^22^ resulting in 3 or 4 control days depending on the month. We fitted conditional logistic regression models for the matched datasets,^23^ with binary responses coded as 1 (case day) or 0 (control day). NO_2_ was included as a five-day moving average, representing the daily mean NO_2_ concentration averaged over the day of admission and four days prior (lag 0–4), based on previous evidence.^24^,^25^ Individual lags of the same day (lag 0) and four days prior (lag 1 to lag 4) were also explored, and the results are presented in Supplementary Materials.

The models were controlled for the daily mean temperature and relative humidity with the same delayed effect (lag 0–4). We assessed the potential nonlinearity of the response functions for meteorological covariates using natural cubic splines with three degrees of freedom. Binary terms for public holidays and weekly consultation rates for influenza were also included. All results were reported as odds ratios (OR) with 95% confidence intervals (CIs) per 10 µg/m^3^ increase in the NO_2_ concentration.

We explored possible effect modifications in stratified analysis by age (0–4, 5–9, and 10–14 years), sex (female and male), season (cold season [defined as January to March and October to December], and warm season [defined as April to September]). We also tested area-level characteristics, including the income deprivation quintile (quintiles of the proportion of population claiming income-related benefits due to being out-of-work or having low earnings from the English Index of Multiple Deprivation 2015) and region (nine regions of England) (Figure S1). We used the Wald test to compare the differences between subgroups of each potential effect modifier.

Preliminary analysis showed that the distance between the patient address and the hospital of admission varied from a few meters to 498 kilometres (Table S1, Figure S2). As people are unlikely to travel hundreds of kilometres for emergency hospital admissions, it is likely that some patients were travelling or staying away from their home addresses at the time of admission, or the patients used an address other than their current address. Therefore, assigning exposure to patients’ registered home addresses may not reflect true exposure. To minimise misclassification of exposure, we applied a threshold for the “patient-to-hospital” distance of ten kilometres, based on previous evidence that 70% of emergency admissions in England happen within 6.2 miles (~ ten kilometres) of a patient’s home.^26^ If the distance between residential postcode and hospital was less than or equal to ten kilometres, we assigned NO_2_ exposure based on the registered patient residential postcode; if the distance was larger than ten kilometres, we assigned NO_2_ exposure based on hospital address. As part of the sensitivity analysis, we tested the validity of the approach by restricting the analysis to only those cases with a patient-to-hospital distance of less than or equal to ten kilometres. This study was conducted in accordance with the guidelines of Strengthening the Reporting of Observational Studies in Epidemiology (STROBE; Table S2). All analyses were performed using R statistical software (v.3.5.1) with the survival package to fit conditional logistic regression models.

## Results

### Descriptive Statistics

**Figure 1:**
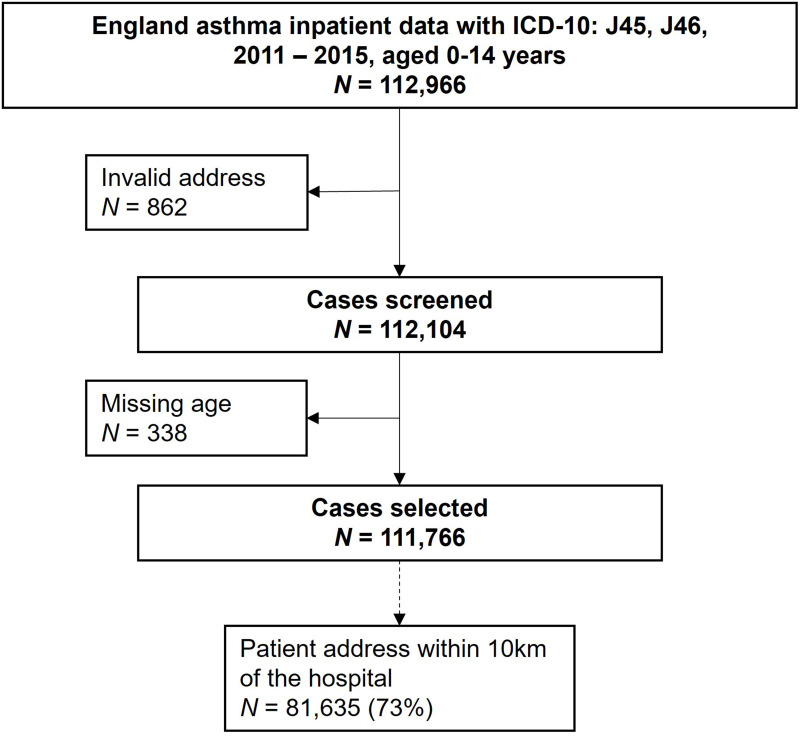
Flow chart of case selection.

After removing 1200 cases with missing information (sex or postcode), we included 111,766 asthma-related emergency hospital admissions in children younger than 15 years residing in England at the time of admission during the five-year study period (Figure 1). Table 1 summarises the characteristics of the emergency admissions included. The mean count of emergency admissions was 61 cases per day (standard deviation 28). The cases were predominantly male (63%) and in younger age groups (43% aged 0–4 years, 37% aged 5–9 years, and 20% aged 10–14 years). 53% of emergency hospital admissions occurred during the cold season. The most deprived areas (the highest quintile of income deprivation) had the highest number of emergency admissions (35%), with admissions gradually decreasing with decreasing income deprivation to 11% of admissions in the lowest income deprivation quintile. For the nine regions in England, over 20% of the asthma emergency admissions occurred in the North West, followed by London (16%), the West Midlands (15%), and the South East (12%). 73% of admissions were within the patient-to-hospital threshold of ten kilometres and we did not observe a temporal pattern in admissions within this threshold (Table S1).

**Table 1:** Characteristics of Asthma Emergency Hospital Admissions of Children Aged 0–14 in England between 2011 and 2015

	Asthma-Related Hospital Admission (Age 0–14 Years)		%Total Population Aged 0–14 of England^a^
	n = 111,766	%	
**Age Group (Years)**			
0–4	47,771	42.8	35.4
5–9	41,161	36.8	31.7
10–14	22,834	20.4	32.9
**Sex**			
Male	70,106	62.7	51.2
Female	41,660	37.3	48.8
**Season^b^**			
Warm	52,835	47.3	–
Cold	58,931	52.7	–
**Income Deprivation Quintile^c^**			
1 (least deprived)	12,489	11.2	18.9
2	15,290	13.7	18.1
3	18,776	16.8	18.6
4	25,923	23.2	20.4
5 (most deprived)	39,288	35.1	24.0
**Region of England**			
South West	8275	7.4	10.0
Yorkshire and the Humber	10,592	9.5	9.8
East Midlands	6143	5.5	8.6
London	18,185	16.3	15.9
North West	22,905	20.5	13.1
West Midlands	16,393	14.7	10.5
East of England	9638	8.6	11.1
South East	13,873	12.4	16.3
North East	5762	5.1	4.7
**Patient-to-hospital Distance**			
≤10 kilometres	81,635	73.0	–
>10 kilometres	30,131	27.0	–

**Notes**: ^a^Based on 2011 census, data source: Office for National Statistics (www.ons.gov.uk). ^b^Cold season: October to March; Warm season: April to September. ^c^Based on Index of Multiple Deprivation 2015, Income Domain.

**Figure 2:**
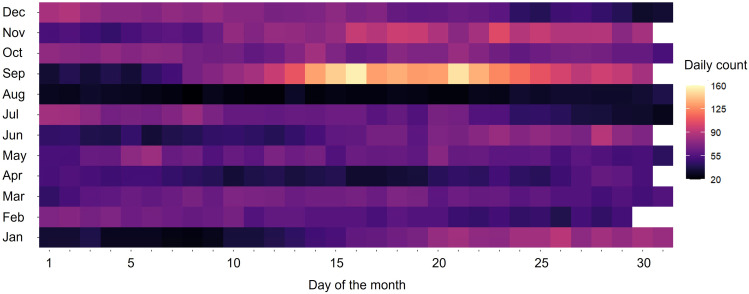
Daily average count of asthma-related emergency hospital admissions in children in England by month, averaged across 2011–2015.

There was no clear seasonal pattern of daily emergency hospital admissions, except a peak in September observed in all years, coinciding with the return to school after the summer holiday (Figure 2). The mean daily NO_2_ exposure across all patients was 19.0 µg/m^3^ (interquartile range (IQR): 12.6–26.5 µg/m^3^) (Table S3). The average NO_2_ exposure showed a clear seasonal pattern (Figure S3), with the cold season (19.6 µg/m^3^) 2.2 µg/m^3^ higher than the warm season (17.4 µg/m^3^) (Table S3). Patients residing in London had the highest daily average NO_2_ exposure (29.5 µg/m^3^), and patients residing in the South West had the lowest (13.0 µg/m^3^) (Table S3). Patients residing in other regions had daily average exposures between 14.6 µg/m^3^ and 19.7 µg/m^3^.

### Associations Between NO_2_ and Emergency Admissions for Asthma

**Figure 3:**
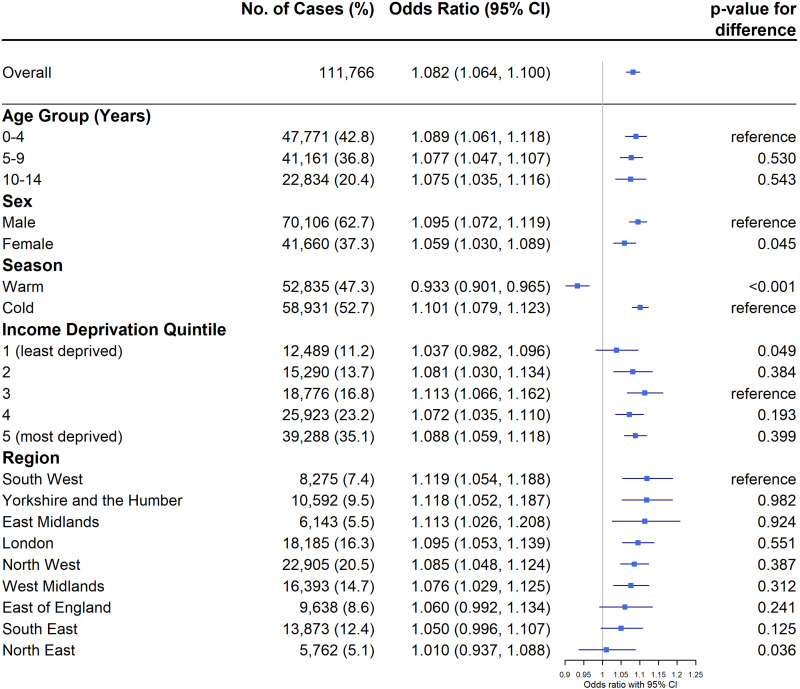
Odds ratios and 95% confidence intervals for asthma-related emergency hospital admissions in children associated per 10 μg/m^3^ increase in NO_2_ (lag 0–4), including overall effect size and stratification by age, sex, season, income deprivation quintile, and region. *P*-value for difference was calculated using a Wald test to compare the difference between subgroups.

We found an 8% increase in the odds of asthma-related emergency hospital admissions in children aged 0 to 14 years per 10 µg/m^3^ increase in five-day moving average NO_2_ (OR 1.08 [95% CI: 1.06–1.10]), after adjustment for time-variant confounders (Figure 3). Effect sizes were larger for male (OR 1.10 [95% CI 1.07–1.12]) than for female (OR 1.06 [95% CI 1.03–1.09], *p*-value for difference 0.045). In age-stratified analyses, we observed similar effect sizes for the three age groups (OR 1.09 [95% CI 1.06–1.12] for 0 to 4 years; OR 1.08 [95% CI 1.05–1.11] for 5 to 9 years; and OR 1.08 [95% CI 1.04–1.12] for 10 to 14 years). There was a difference in the direction of association between warm and cold seasons (Figure 3), with a 10% increase in odds during cold season (OR 1.10 [95% CI 1.08–1.12]) but 7% decrease during warm season (OR 0.93 [95% CI 0.90–0.97]). Stratified analysis by area-level income deprivation showed no clear trend. The highest effect was observed in the third deprivation quintile (OR 1.11 [95% CI 1.06–1.16]) and the lowest in the least deprived quintile (OR 1.04 [95% CI 0.98–1.10]). Effect sizes varied by region, with the highest observed in the South West (OR 1.12 [95% CI 1.05–1.19] and lowest in the North East (OR 1.01 [95% CI 0.94–1.09]). The North East also had the lowest number of asthma-related emergency admissions. Notably, East Midlands had a comparatively high effect estimate (OR 1.11 [95% CI 1.03–1.21]), but the second lowest number of asthma admissions.

### Sensitivity Analyses

In sensitivity analyses, restricting admissions to those within the ten-kilometre patient-to-hospital distance threshold, we found similar effect sizes compared to the main analysis, although with larger confidence intervals due to smaller sample sizes (Table S4). In contrast to the main analysis, we did not observe a decrease in odds during the warm season (OR 1.07 [95% CI 0.99–1.15], *p* > 0.05). Findings from the single-day lag analysis were generally consistent with those using mean lag 0–4 (Tables S5–S7), but the odds were moderately larger for mean lag 0–4 than for single-day lags.

## Discussion

In this case-crossover study, we investigated the association between daily ambient NO_2_ exposure and asthma-related emergency hospital admissions among children aged 0–14 years in England. NO_2_ exposure was measured cumulatively over the case day and four days prior (mean lag 0–4). We found positive associations between NO_2_ and all asthma emergency hospital admissions and for each age and sex strata examined.

The effect estimates were similar across the three age groups, but we observed an effect modification by sex. There were noticeably more male than female asthma hospital admissions in children, which is consistent with the results of previous studies.^27–29^ We also found that the association between short-term exposure to NO_2_ and increased risk of asthma emergency admissions was stronger in male children. However, other studies have reported mixed findings, with higher risk in both male and female children.^30–32^ The reasons for this difference remain unclear, although studies have suggested factors including sex-linked physiological differences such as growth rates and airway sizes, and social differences such as activity patterns.^33–35^

We observed seasonality in emergency hospital admissions for asthma in children. In this study, short-term exposure to NO_2_ during the cold season was statistically significantly associated with a 10.1% increased risk of asthma emergency admissions per 10 µg/m^3^ increase in NO_2_ exposure. There was no clear evidence of an association in the warm season based on the sensitivity analysis. In England, air pollution levels were generally higher during the cold season than during the warm season (Figure S3). Therefore, air pollution could be a key asthma exacerbation factor during the cold season, whereas other factors, such as pollen, play a more important role in the warm season. Moreover, cold air can trigger functional changes in the airways,^36^ and air pollution may further irritate the lungs during the cold season, as it passes through the upper respiratory tract to the deep-end bronchus. In contrast to our findings, several previously published studies have found stronger associations among children during the warm season.^2^,^24^,^37^ This difference might be related to the different study characteristics, as these studies used population-level (or group-level) exposure rather than individual-level exposure and assessed emergency department visits which are likely to be less severe than emergency admissions.^38^

The observed peak in asthma-related admissions in September is likely linked to the known “September asthma epidemic”, when most children return to school in England. There are several potential reasons for this. First, school children may be re-exposed to respiratory viral tract infections (especially rhinovirus) after the summer holiday, and indoor mixing may lead to asthma exacerbations.^39^ Second, the school environment around August and September exhibited relatively high levels of allergens such as pollen and mould spores.^40^ Finally, returning to school may lead to high stress, which can worsen asthma symptoms.^40^

The area-level deprivation index used in this study is based on deprivation measures for geographic areas containing approximately 1500 people, and therefore may not reflect the socioeconomic status of individuals or families. This may explain why the deprivation stratification analysis did not show a consistent effect among the least to most deprived quintiles. Although there is evidence that more deprived households tend to live in areas with poor air quality,^41^ one study found that low socioeconomic status was not associated with asthma prevalence in England.^42^ The number of asthma emergency admissions is generally proportional to the population in each region. However, it is notable that 13.1% of children aged 0 to 14 years in England live in the North West, yet 20.5% of asthma emergency admissions came from this region. This may be linked to the relatively high daily average NO_2_ concentrations in the region (the second highest region after London). The four regions that showed statistically significant associations in both the primary and sensitivity analyses were North West, London, East Midlands, and South West. However, the effect estimates did not show a significant disparity across these regions.

Most previous studies on the association between air pollution and hospital admissions or visits used daily counts of hospital admissions or visits in a Poisson regression time-series or case-crossover analysis.^4^,^19^,^43–47^ These studies account for temporal changes in exposure but assign the same exposure estimate to all cases in the study area, and therefore do not account for spatial differences in exposure. Owing to data requirements (ie individual addresses and pollution estimates), few studies have examined associations at the individual level,^17^,^32^,^48^,^49^ and less evidence is based on estimates from detailed exposure models.^12^,^14^,^50^ We identified one study that investigated the association between NO_2_ and asthma hospitalisation among socioeconomically disadvantaged populations (< 65 years of age) in the United States using exposure estimated from machine learning-based models.^14^ The study found that a 1-ppb (equivalent to around 1.88 µg/m^3^) increase in NO_2_ at lag 0–6 was associated with 0.31% (95% CI 0.24–0.37%) increase in the risk of asthma hospitalisation. Children were at a higher risk than the overall population, with 0.35% (95% CI 0.27–0.44%) for 0–4 years and 0.52% (95% CI 0.43–0.62%) for 5–12 years. Additionally, for the study population of all ages, a higher risk was observed in inpatients living in more disadvantaged communities with a high area-level deprivation index (0.33% [95% CI 0.25–0.42%]) than in the medium to low index (0.26% [95% CI 0.20–0.31%]). No modification effect was found between male and female.

Although not directly comparable, our results are in agreement with those of several population-level studies that have assessed NO_2_ and asthma among children.^25^,^51^ For example, a study in Canada reported a 9% increased risk per 13.5 ppb (equivalent to approximately 25.4 µg/m^3^) increase in asthma emergency visits among children aged 5 to 14 years using a mean lag 0–4.^24^ The finding is similar to a study in Italy, where a 22.3 µg/m^3^ increase in NO_2_ was associated with a 10.7% increase in asthma admissions for children aged 0 to 14 years.^16^ The effect estimate in our study was similar in magnitude (8.2%), but the increment level of NO_2_ was 10 µg/m^3^, suggesting a higher risk.

### Strengths and Limitations

A major strength of this study is the use of highly detailed modelled air pollution data that made it possible to assign NO_2_ estimates to patients’ residential postcodes, rather than using measurement data from the nearest monitoring stations, as in previous studies, which can lead to substantial measurement errors. Measurement errors existed in both the monitored and modelled pollutant concentrations. Errors in exposure models could be linked to a lack of representation of the sources and instrument data used to calibrate the model. The extent of air pollution measurement errors could have an impact on epidemiological studies, especially the statistical power to detect effects. However, studies with only concentrations from scattered monitoring stations have more bias, which increase the likelihood of obtaining a null association in the odds ratio.^52^ Various simulation studies have suggested that the attenuation of health effect estimates in time-series studies may result from misspecification of exposure.^53–56^

Some limitations of our study should be noted. First, our definition of asthma-related emergency hospital admission required an ICD code for asthma as the primary diagnosis at admission. This approach has been shown to be highly specific for identifying children with a documented diagnosis of asthma, but it excludes children with undiagnosed asthma which is common, particularly before the age of 6 years, when spirometry can be performed. Therefore, these findings cannot be extrapolated to a cohort of children who present with asthma symptoms (eg, cough, wheezing, or dyspnoea) in the absence of a pre-existing diagnosis of asthma. Second, indoor air pollution, from sources such as smoking and cooking, can also irritate the airways and lead to respiratory diseases.^57–59^ However, due to the individual-level nature and the scale of the study, we were not able to consider indoor air pollution in our analysis. Third, it is not certain whether the patients were at or around their home addresses during the five-day cumulative exposure window. Although we adopted a “patient-to-hospital” distance with a threshold of ten kilometres, the results were still subject to exposure misclassification. The effect estimates for NO_2_ in the sensitivity analysis, using only cases within the ten-kilometre threshold, did not differ substantially from the primary analysis, except for the null association during the warm season. This suggests that the approach of assigning exposure based on the distance threshold can be applied in future studies, yet the threshold needs to be carefully chosen and justified. One may consider using different distances for rural and urban locations. Fourth, some modifiers (eg, deprivation quintile) were only available for small geographies, meaning that some findings may not truly reflect individuals within that area. The lack of more granular data (eg, individual socioeconomic status) may explain why we did not find a modification effect in area-level analyses. Fifth, stratification in the analysis to examine effect sizes by subgroup may have resulted in small numbers and reduced statistical power. Last, because of the complexity of modelling and linking exposure from the spatio-temporal air pollution model to the health data, we only conducted a single-pollution analysis. Multi-pollutant models often face validity issues due to multicollinearity, particularly when the assessed pollutants are highly correlated.^60^ Nonetheless, humans experience simultaneous exposure to a diverse array of other air pollutants (eg, particulate matter) and allergens (eg, pollen and spores).^61^ Adopting a multi-pollutant approach may offer a more realistic representation of the environmental conditions individuals may experience, and mitigating the risk of confounding in epidemiological studies.

## Conclusions

Our findings suggest a significant increase in the risk of asthma-related emergency admissions with higher levels of NO_2_ exposure in children in England, particularly during colder seasons and among males. Future guidance and policies should consider incorporating proven modifications, such as using season-specific countermeasures for air pollution control, to protect at-risk populations. Further research is needed to explore the potential interaction between air pollutants, allergens, and other factors (eg, ethnicity and underlying health conditions) that could exacerbate childhood asthma and to inform targeted public health strategies to minimise these risks.

## Data Sharing Statement

Data may be obtained from a third party and are not publicly available. The UK Small Area Health Statistics Unit (SAHSU) does not have permission to supply data to third parties.
